# A Systematic Review of the Natural Virome of *Anopheles* Mosquitoes

**DOI:** 10.3390/v10050222

**Published:** 2018-04-25

**Authors:** Ferdinand Nanfack Minkeu, Kenneth D. Vernick

**Affiliations:** 1 Institut Pasteur, Unit of Genetics and Genomics of Insect Vectors, Department of Parasites and Insect Vectors, 28 rue du Docteur Roux, 75015 Paris, France; ferdinand.nanfack-minkeu@pasteur.fr; 2 CNRS, Unit of Evolutionary Genomics, Modeling and Health (UMR2000), 28 rue du Docteur Roux, 75015 Paris, France; 3 Graduate School of Life Sciences ED515, Sorbonne Universities, UPMC Paris VI, 75252 Paris, France

**Keywords:** insect vectors, virome, arbovirus, insect immunity, host–pathogen interactions, malaria

## Abstract

*Anopheles* mosquitoes are vectors of human malaria, but they also harbor viruses, collectively termed the virome. The *Anopheles* virome is relatively poorly studied, and the number and function of viruses are unknown. Only the o’nyong-nyong arbovirus (ONNV) is known to be consistently transmitted to vertebrates by *Anopheles* mosquitoes. A systematic literature review searched four databases: PubMed, Web of Science, Scopus, and Lissa. In addition, online and print resources were searched manually. The searches yielded 259 records. After screening for eligibility criteria, we found at least 51 viruses reported in *Anopheles*, including viruses with potential to cause febrile disease if transmitted to humans or other vertebrates. Studies to date have not provided evidence that *Anopheles* consistently transmit and maintain arboviruses other than ONNV. However, anthropophilic *Anopheles* vectors of malaria are constantly exposed to arboviruses in human bloodmeals. It is possible that in malaria-endemic zones, febrile symptoms may be commonly misdiagnosed. It is also possible that anophelines may be inherently less competent arbovirus vectors than culicines, but if true, the biological basis would warrant further study. This systematic review contributes a context to characterize the biology, knowledge gaps, and potential public health risk of *Anopheles* viruses.

## 1. Introduction

*Anopheles* mosquitoes are the vectors of human malaria, which causes at least 400,000 deaths and 200 million cases per year [1]. Approximately 90% of malaria deaths occur in sub-Saharan Africa, 7% in South-East Asia and 2% in the Eastern Mediterranean Region, with children under five years of age the most affected. More than 480 species of *Anopheles* have been described worldwide and about 70 of these are responsible for human malaria transmission, with about 40 regarded as the dominant malaria vector species [2,3].

However, the research focus on *Anopheles* as vectors of malaria has led to a relative lack of study about *Anopheles* viruses. In addition to malaria parasites, *Anopheles* mosquitoes also harbor viruses, collectively termed the virome. The *Anopheles* virome is poorly studied, and the number and function of viruses are unknown. Some of them are confirmed arthropod-borne pathogenic viruses (arboviruses), which multiply in the mosquito vector before transmission to a vertebrate host. Others are thought to be insect-specific viruses that may replicate only in the insect host [4,5].

Culicine mosquitoes such as *Aedes* and *Culex* are the main vector of arboviruses such as dengue virus (DENV; genus *Flavivirus*, family *Flaviviridae*), yellow fever virus (YFV, genus *Flavivirus*, family *Flaviviridae*), chikungunya (CHIKV, genus *Alphavirus*, family *Togaviridae*), and others. Only one arbovirus is known to be consistently transmitted by *Anopheles* mosquitoes, the alphavirus o’nyong-nyong (ONNV, genus *Alphavirus*, family *Togaviridae*) [6,7,8,9], which is closely related to CHIKV [10].

RNA viruses from various families (e.g., *Flaviviridae*, *Togaviridae*, *Peribunyaviridae*, *Rhabdoviridae*, *Mesoniviridae*, *Reoviridae*, and *Dicistroviridae*) and the taxon *Negevirus* have been described in *Anopheles* mosquitoes [5,11,12,13,14,15]. These viruses have been discovered by isolation from cell cultures, by reverse transcriptase PCR (RT-PCR) and manual sequencing targeting regions of known viruses, or by deep sequencing of field-caught insect samples [16,17].

In addition to ONNV, other viruses with potential to cause febrile disease if transmitted to humans or other vertebrates have been isolated from *Anopheles*, including Nyando virus [18,19], Batai virus [20], Japanese encephalitis virus [21], Myxoma virus [22], and West Nile virus [23]. During a recent epidemic, Rift Valley fever virus (RVFV) was present in *Anopheles* females, males, and larvae, indicating vertical transmission [24,25].

Studies to date have not provided evidence that *Anopheles* can contribute to the transmission and maintenance of any of these arboviruses, other than ONNV. However, anthropophilic *Anopheles* vectors of malaria are also constantly exposed to arboviruses in infected human bloodmeals. It is possible that in malaria-endemic zones, febrile symptoms of malaria may mask symptoms of arbovirus infection and cause misdiagnosis. It is also possible that, for an unknown reason, anophelines are less competent arbovirus vectors than culicines, but if true, this would be biologically interesting, and would warrant further study. Mechanisms of *Anopheles* anti-viral immunity have been little examined [26,27,28]. The relative lack of virus transmission by *Anopheles*, if true, is puzzling because *Anopheles*, especially highly anthropophilic taxa, would seem well-placed to serve as intermediaries for virus spillover from other vertebrates to humans [29], as well as vectors for maintenance of transmission.

The characteristics of *Anopheles* viruses that comprise the natural virome flora are also poorly characterized, thereby creating little appreciation of their number and significance. Examination of the literature suggests that the number of *Anopheles* viruses is underestimated, including potentially pathogenic arboviruses. Here, we carry out a systematic literature search in order to summarize as comprehensively as possible the known viruses of *Anopheles* mosquitoes.

## 2. Materials and Methods

Four databases were searched in this work: PubMed, Web of Science, Scopus, and Lissa. In addition, manual searches of online and print resources were carried out.

Three combinations of keywords were used for searching the PubMed database. These were (i) ((“anopheles”[Title] OR “anopheles”[All Fields]) AND (viruses[Title]) NOT (Aedes[Title] OR Culex[Title])); (ii) (((Anopheles AND viruses) NOT (Aedes OR Culex)) OR ((“Anopheles/virology”[Mesh]) NOT (“Culex”[Mesh])) NOT (“Aedes”[Mesh]))); and (iii) ((Anopheles viruses [Title]) NOT (Aedes [Title] OR Culex [Title])).

Searching article titles proved to be useful because the search terms were compatible across bibliographic databases. Thus, search (iii) above was easy to translate into the Web of Science, Scopus, and Lissa databases, as follows. Web of Science advanced search of all databases, including Web of Science Core Collection, KCI-Korean Journal Database, MEDLINE, Russian Science Citation Index and SciElo Citation Index: TI = Anopheles AND TI = Viruses NOT TI = (Aedes OR Culex); Scopus advanced search: (TITLE (Anopheles AND Viruses) AND NOT TITLE (Aedes OR Culex)); and the French language Lissa: ‘Virus d’Anopheles.ti SAUF (Aedes OR Culex).ti’’.

The above searches were carried out from 15 January to 4 May 2016. Moreover, an alert with the keyword (((Anopheles AND viruses) NOT (Aedes OR Culex))) OR (((“Anopheles/virology”[Mesh]) NOT “Culex”[Mesh]) NOT “Aedes”[Mesh]) was created and followed in PubMed from 15 January 2016 to the submission date of this manuscript. Anopheles virus/virus d’anophèles were also searched in Google. In addition, the key word ‘’Anopheles’’ was used to identify arboviruses associated with *Anopheles* species in the online Arbocat Arbovirus Catalog resource, maintained by the Centers for Disease Control and Prevention, USA (https://wwwn.cdc.gov/arbocat/). In addition to these online searches, the books from the central library of the Institut Pasteur (Scientific Media and Information Center (CeRIS)) specialized in microbiology, virology, entomology, immunology, molecular biology, and biochemistry were searched.

Finally, eligibility criteria were applied for inclusion of a virus in the study: (i) virus species and name coherent with the standards of the International Committee on Taxonomy of Viruses (ICTV), (ii) reporting of diagnostic tools to permit independent detection, and (iii) some amount of nucleotide sequence. Reports of a putative virus were ineligible if they met none of these criteria, for example if the report was based only on observation of cytopathic effects on cultured cell lines, or pathology in mice.

## 3. Results

### 3.1. Bibliographic Search of Publication History on Anopheles Viruses

The search terms ((Anopheles viruses [Title]) NOT (Aedes [Title] OR Culex [Title])) yielded 36, 43 and 58 records in PubMed, Scopus, and Web of Science databases, respectively, on 4 May 2016 (PRISMA Flow Diagram, Figure S1). The 36 articles found in PubMed produced meaningful outputs, corresponding to recent reports of *Anopheles* virus. For example, in PubMed, the article number PMID: 27138938 (published 3 May 2016) was returned, but in Web of Science and Scopus the most recent articles were published in November 2015.

The alerts in PubMed using criteria (((Anopheles AND viruses) NOT (Aedes OR Culex))) OR (((“Anopheles/virology”[Mesh]) NOT “Culex”[Mesh]) NOT “Aedes”[Mesh]) yielded 14 articles between 15 January and 18 August 2016. These articles have the following numbers: PMID: 26807720, PMID: 25882523, PMID: 26821654, PMID: 25879960, PMID: 25637950, PMID: 26492074, PMID: 26416112, PMID: 26271277, PMID: 27113956, PMID: 25222233, PMID: 27138938, PMID: 26401843, PMID: 27456078, PMID: 26807720. Only the six articles in bold among these 14 correspond to *Anopheles* viruses.

The Lissa database of scientific literature written in French returned a single article using the above English search terms. However, this article in French (with English keywords) was written in 1957, and was not included in any of the three other databases. In addition, when using French language search terms, the Lissa database identified 13 additional articles (for a total of 14), all in French, that were uniquely identified by Lissa and not by the other three databases. Nevertheless, some of the search terms such as “virus” and “Anopheles” are spelled the same in French and English, and further work would be required to determine whether the different search results are due to search term language, or distinct database contents.

### 3.2. Viruses

**Figure 1:**
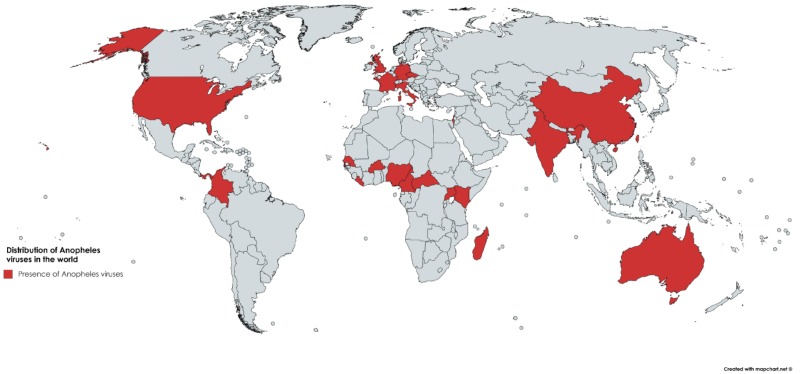
Global distribution of reported *Anopheles* viruses by country (red, countries with viruses in *Anopheles*).

Both DNA and RNA viruses have been reported infecting *Anopheles* species, although reports of RNA viruses are more prevalent. RNA viruses include *Alphavirus*, *Phlebovirus, Flavivirus, Orthobunyavirus, Dicistrovirus, Cypovirus, Mononegavirus, Totivirus*, and *Orbivirus* genera. DNA viruses include *Densovirus*, *Poxvirus*, *Iridovirus* (Table 1). *Anopheles* viruses have been reported on all continents except the poles (Figure 1).

### 3.3. DNA Viruses

#### 3.3.1. Densovirus: *Anopheles gambiae* Densovirus (AgDNV)

The densoviruses belong to the *Parvoviridae* family, characterized by a non-enveloped virion containing a linear single-stranded DNA genome. AgDNV has a genome size of 4139 nt and is organized as two overlapping reading frames that encode the viral proteins (VP) of which activity is fundamental for virus infectivity and two non-structural (NS) proteins involved in the DNA replication. The NS1 portion displays 87% homology with *Aedes aegypti* densovirus (AeDNV) [33].

AgDNV was discovered in the *An. gambiae* cell line, Sua5B, during an experiment to infect the cells with *Wolbachia*. It was maintained between different generations by vertical and horizontal transmission and has no detectable effect on mortality of *An. gambiae* larvae. Virus purification was done from crude cell lysates on a density gradient, and icosahedral, non-enveloped particles of 20 nm were observed by transmission electron microscopy [33].

The use of DNVs as expression vectors was demonstrated by the transfection of *Aedes albopictus* C6/36 cell line with a plasmid containing an infectious sequence of *Ae. aegypti* DNV. This infection yielded the same quantity of the monomeric replicative form as infection with wild type virions [64]. A recombinant AgDNV carrying the enhanced green fluorescent protein (EGFP) under control of actin5C promoter has been produced [33]. EGFP transducing virions infected 50% of adults and were able to disseminate to the fat body, midgut, hindgut, malpighian tubules, and ovaries, and were vertically transmitted to subsequent generations. These results indicate that recombinant AgDNV could be a candidate for paratransgenesis, for example by carrying an anti-*Plasmodium* peptide for reducing the vector competence of *Anopheles* to *Plasmodium* spp. In addition, the AgDNV titer is higher in older *An. gambiae* adults compare to the larvae and pupae stages, suggesting that it could have potential as an adult stage bio-insecticide [65]. Tested DNVs are innocuous to mammals [64].

#### 3.3.2. Iridovirus: *Anopheles minimus* Virus (AMIV)

*Anopheles minimus* virus is an iridescent virus (IIV) belonging to *Iridovirus* genus (family, *Iridoviridae*). The genera *Chloriridovirus, Lymphocystivirus, Megalocytivirus,* and *Ranavirus* are also included in this virus family. The icosahedral virions of AMIV are roughly 130 nm in diameter and the DNA genome is 163 kb in size [66]. The genomic DNA is associated with proteins and the internal membrane is composed of phospholipids. Circularly permuted double-stranded DNA (dsDNA) genomes are characteristic of this family.

AMIV was isolated from wild adult *Anopheles minimus*, a major Southeast Asian malaria vector, after inoculation of C6/36 cells with mosquito extract [35]. BHK21 and Vero-E6 cells can also be infected with AMIV, with a significant cytopathic effect [66]. AMIV is the first iridovirus isolated from *Anopheles* species. An *Ae. aegypti* iridovirus (IIV-6) causes cytopathic damage leading to the reduction of body size, fecundity and longevity. Horizontal transmission by cannibalism and vertical transmission of IIV-3 were observed in *Ochlerotatus taeniorhynchus* [67]. Iridovirus infection leads to an apoptotic response in invertebrate and vertebrate cells [67].

#### 3.3.3. Poxvirus: Myxoma Virus (MYXV)

The myxoma virus genome was detected by polymerase chain reaction (PCR) and sequencing in wild caught *An. maculipennis* that fed on wild rabbits [22]. The *An. maculipennis* group includes major historic vectors of human malaria in North African and Europe [68]. Myxoma virus is in the *Leporipoxvirus* genus and *Poxviridae* family. This family includes the subfamilies *Chordopoxvirinae* and *Entomopoxvirinae*. The latter infects insects and comprises *Alphaentomopoxvirus*, *Betaentomopoxvirus*, and *Gammaentomopoxvirus* genera. Myxoma virus can be mechanically transmitted by mosquitoes and fleas but does not replicate in them, and thus is not an arbovirus. The *Poxviridae* genome is comprised of linear dsDNA, and is enveloped. Poxviruses share features with other DNA viruses such as the asfarviruses, iridoviruses, and phycodnaviruses [69].

### 3.4. RNA Viruses

#### 3.4.1. Alphavirus: O’nyong Nyong Virus (ONNV), Venezuelan Equine Encephalitis Virus (VEEV), Western Equine Encephalitis Virus (WEEV), Sindbis Virus (SINV), Semliki Forest Virus (SFV), and Eilat Virus (EILV)

The *Alphavirus* genus in the *Togaviridae* family is frequently associated with *Anopheles* mosquitoes. O’nyong nyong virus (ONNV) is generally regarded as the only arbovirus transmitted by *Anopheles* mosquitoes. Alphaviruses are enveloped viruses with a single-stranded RNA of positive sense and a genome size of around 10,000 nucleotides. The 5′ end is capped and the 3′ end is polyadenylated. The linear RNA genome encodes nonstructural proteins (nsP1 to nsP-4), and structural proteins, although only the nsPs are translated from the genome, while the structural proteins are translated from a subgenomic RNA transcribed after infection. The nonstructural protein nsP1 is necessary for infectivity, nsP2 is necessary for replication and transcription of viral RNAs, nsP3 forms cytoplasmic complexes with different host factors, and nsP4 is a RNA-dependent RNA polymerase. A domain-swap experiment replacing part of the CHIKV nsP3 with the ONNV sequence allowed the chimeric virus to infect *An. gambiae* [70], highlighting a determinant of mosquito host specificity that requires further investigation. The capsid protein, the glycoproteins (E1 and E2) and the small peptides, E3 and 6K are structural proteins. A single mutation of E1 glycoprotein (E1-A226V) increases CHIKV transmission by the mosquito *Aedes albopictus* [71]. E2 is involved in antigenicity and viral pathogenesis [72]. The E3 peptide is necessary for protein heterodimerization, and the deletion of the 6K peptide in Ross River Virus genome reduces pathogenicity and viral titer in mice [73]. The interactions between the structural proteins are also indispensable for virion integrity and virus assembly [32,74].

The common symptoms of ONNV in humans are fever, rash, headache, polyarthritis-like illness, and back pains, but the infection is often asymptomatic. An ONNV epidemic in Uganda, Tanzania, Kenya, Malawi, Senegal, Democratic Republic of Congo infected approximately 2 million people, but no fatal cases were reported [7,18]. Another outbreak of ONNV was reported in Uganda in 1996, with morbidity rates of 45–65% in some villages. ONNV was detected by serologic test and quantitative RT-PCR in 26% of Liberian refugees tested in 2003 [8,9]. The virus was detected in a pool of wild *Anopheles funestus* and *An. gambiae* collected in Uganda and Kisumu, Kenya by inoculation of a suspension into albino Swiss mice, and independently also from patient sera [8,18,50]. Antibodies against ONNV found in the sera of inoculated mice suggested that the virus was pathogenic.

The important African malaria vectors, *An. funestus* and *An. gambiae,* are also able to transmit ONNV to mice [75,76]. The RNA interference (RNAi) pathway is necessary for protection of *An. gambiae* against ONNV infection [27]. However, the RNAi pathway displays ONNV antiviral activity in *An. gambiae* only during the disseminated systemic infection, and not in the primary midgut infection by bloodmeal [26]. The immune pathways Janus kinases/Signal Transducer and Activator of Transcription (JAK/STAT) and Immune Deficiency (Imd) display a reciprocal effect, as ONNV antiviral mechanisms in the primary midgut infection but not against the disseminated infection in the hemocoel.

Venezuelan equine encephalitis virus (VEEV) is an arbovirus in the Americas, where numerous outbreaks have been reported with equine and human deaths [77]. A large epidemic of VEEV in Colombia in 1995 caused more than 70,000 human cases and 300 deaths [78]. VEEV was isolated from two pools of *Anopheles p*. *pseudopunctipennis* in Mexico in 1972, was cultured in C6/36 cells, and an infectious clone was generated [60]. Western equine encephalitis virus (WEEV) is a recombinant virus that can infect humans and other vertebrates, with a capsid protein related to Eastern equine encephalitis virus (EEEV) whereas the glycoprotein sequences are closer to Sindbis virus [79]. An epizootic occurred in Argentina in 1982, and more than 150,000 mosquitoes of different genera were collected by Centers for Disease Control (CDC) light traps between 1982 and 1983 [63]. From these mosquitoes, WEEV was isolated from a pool of *An. albitarsis* inoculated on Vero cells [63].

*An. albimanus* mosquitoes were competent for infection with Sindbis virus (SINV) by feeding on infected rabbit blood, displaying an infection prevalence of 64%, as well as the ability to transmit the virus to baby chicks [80]. SINV was also isolated from a pool of *Anopheles* spp. collected in China in 1990 and was used to generate an infectious clone [53]. SINV was first isolated in Sindbis village in Egypt in 1952 in *Culex* spp., but *Anopheles* mosquitoes seem also able to transmit this virus [80].

Semliki Forest virus (SFV) was discovered in *Aedes abnormalis* collected in Uganda in 1942 [27,81]. Multiple *Anopheles* species (*An. stephensi*, *An. freeborni*, *An. sundaicus* and *An. labranchia*) were infected with SFV by membrane feeding [82]. In an outbreak in Bangui, Central African Republic in 1987, SFV was isolated from patient sera and from pools of *Ae. africanus, Ae. aegypti, An. coustani,* and *An. funestus* [52].

Eilat virus (EILV) was isolated from a pool of *An. coustani* mosquitoes and displays inability to replicate in the vertebrate cellular environment [13,43]. EILV was transmitted by bloodfeeding to *An. gambiae*, *C. quinquefasciatus*, and *Ae. aegypti* but not to *Ae. albopictus***,** and may be vertically transmitted in the infected species, but the virus was not detected in the ovaries [13]. EILV did not display cytopathic effect in *Aedes* C6/36 and or C7/10 cells despite high replication at 12 h post infection. One proposed hypothesis is that EILV may have secondarily lost the ability to infect vertebrate cells, rather than being insect-specific as an ancestral character.

#### 3.4.2. Flavivirus: West Nile Virus (WNV), Japanese Encephalitis Virus (JEV), Wesselsbron Virus (WSLV), *Anopheles* Flavivirus (AnFV), *Anopheles gambiae* Flavivirus (AngFV), *Anopheles squamosus* Flavivirus (AnsFV), Stratford Virus (STRV), Karumba Virus (KRBV), Haslams Creek Virus (HaCV), Dairy Swamp Virus (DSwV), Mac Peak Virus (McPV), Long Pine Key virus (LPKV), Kampung Karu Virus (KPKV)

Members of the *Flavivirus* genus in the family *Flaviviridae* are generally thought to be transmitted by culicine mosquitoes. However, a number studies have detected flaviviruses in *Anopheles* species, suggesting that anophelines could also be involved in transmission [23]. Flaviviruses are characterized by an enveloped virion carrying a single-stranded RNA genome of positive polarity. The linear genome of 10 to 11 kb is flanked by 5′ and 3′ untranslated regions (UTR) that encode for a single open-reading frame. Translation produces a single polyprotein cleaved in 10 proteins: three structural (C, prM, E), and seven non-structural (NS1, NS2A, NS2B, NS3, NS4A, NS4B, and NS5) proteins [83].

The main reported vectors of West Nile virus (WNV) are *Culex* spp. Transmission is mainly zoonotic but with significant levels of human infection, including occasional mortality [84]. A pool of *An. pauliani* wild unfed females was found to be positive for WNV RNA by RT-PCR in Madagascar [23]. A pool of *An. maculipennis* collected in Italy between 2008 and 2012 was also found positive for WNV RNA by RT-PCR [61]. A pool of *An. maculipennis* collected in Serbia after the 2012 WNV outbreak was also positive for WNV RNA by RT-PCR, which suggested a potential important role for this species in transmission [62].

Japanese encephalitis virus (JEV) is an endemic causative agent of encephalitis in Asia and India. JEV infects humans and other vertebrates such as horses, dogs, and reptiles. Based on envelope protein gene sequences, JEV strains can be classified into five different genotypes, I–V, with specific geographic distributions [85]. Due to the availability of a vaccine, outbreaks of JEV are rare. JEV was detected and isolated from a pool of *Anopheles peditaeniatus* females collected from 1985–1987 in India [45]. JEV was isolated from C6/36 cells inoculated with extract from pools of *Anopheles sinensis* captured by carbon dioxide traps or sweep nets in Taiwan from 2005 to 2012, for a calculated infection rate of 2.3 per 1000 mosquitoes [21]. JEV genotype I was isolated from host-seeking *An. sinensis* collected by aspirator in Japan [46]. *An. sinensis* is a dominant human malaria vector in Asia [2,86].

Wesselsbron virus (WSLV) shares ecological niches and similar livestock symptoms with Rift Valley Fever virus and misdiagnosis is common [34,87]. WSLV RNA was detected using molecular diagnostic tools in Kenya from *An. coustani* and in humans in Senegal [34,87]. *Aedes* mosquitoes are the presumed main vector of WSLV but the involvement of *Anopheles* mosquitoes remains to be elucidated.

*Anopheles* flavivirus (AnFV), the first flavivirus discovered in *Anopheles*, was identified in a population virome survey in Liberia and Senegal [32]. An RT-PCR diagnostic assay confirmed the presence of AnFV RNA in wild *Anopheles*, with a prevalence of 12%. *Anopheles gambiae* flavivirus (AngFV) and *An. squamosus* flavivirus (AnsFV) were detected in *An. gambiae* and *An. squamosus* respectively by a nucleic acid melting-curve analysis from mosquitoes collected in Kenya [34]. AngFVs and AnsFV share 77% of nucleotide identity.

Stratford virus (STRV) RNA was detected in an isolate of *Anopheles annulipes* collected in Australia from 1995–2013 [54]. Sequence analysis based on part of the NS5 gene sequence displayed 95–99% homology among the different isolates of STRV, and 2% divergence was detected between the isolates collected in 1995–2013 and the first isolates of 1961. The main vector of STRV is thought to be *Aedes* spp., without important contribution by *Culex* spp. [54].

A study of *Anopheles* samples collected in Australia identified multiple insect-specific viruses that do not infect vertebrate cells, and display fine species-specific host restriction for the *Anopheles* host in which they were identified [42]. The genome of Karumba virus (KRBV) was obtained from single and pooled mosquitoes collected in two different sites in Australia. In addition, the same authors also discovered Haslams Creek virus (HaCV), Dairy Swamp virus (DSwV), and Mac Peak virus (McPV) in pools of *An. annulipes, An. bancrofti*, and *An. farauti*, respectively [42]. HaCV, DSwV, and McPV failed to replicate in vitro on cell lines C6/36 *Aedes albopictus*, MOS55 *An. gambiae*, ISE6 tick, or S2 *Drosophila melanogaster.* No replication of KRBV was observed on the following vertebrate cells: BSR *Mesocricetus auratus,* Vero *Cercopithecus aethiops,* or DF-1 *Gallus gallus* [42]. The complete genome sequences were assembled by deep sequencing of mosquito RNA. Viral sequences were detected in the 21-nucleotide small RNA fraction, which suggested that double stranded RNA (dsRNA) intermediates produced during viral replication were cleaved by the siRNA pathway into viral RNAs (viRNAs). Presence of viRNAs is evidence of active virus replication. The prevalence of KRBV found in *Anopheles meraukensis* was 91.7% and 100% in Wyndham and Karumba, respectively. The prevalence of KRBV in wild type mosquitoes is quite high and highlights the need to study its impact on *Anopheles* species. Specific RT-PCR assays detected HaCV and McPV RNA in wild *An. annulipes*, *An. bancrofti*, and *An. farauti*.

Long Pine key virus (LPKV) was isolated in the United States from a pool of 50 *An. crucians,* and Kampung Karu virus (KPKV) was isolated from a single *An. tesselatus* in Malaysia [47]. Both viruses were cultured on C6/36 cells and cytopathic effects were observed at 7 d post-inoculation. Inoculation of BHK-21 and Vero cells with LPKV and KPKV produced neither replication nor cytopathic effects, and these viruses did not display pathology in mice [47]. Hemagglutination-inhibition tests for KPKV were not possible due to absence of reactive hemagglutinin. Despite being insect-specific, LPKV and KPKV reacted serologically with antibodies directed against some dual-host flaviviruses such as WNV, JEV, and Dengue virus [47].

#### 3.4.3. Phlebovirus: Rift Valley Fever Virus (RVFV)

Rift Valley fever virus (RVFV) belongs to the *Phlebovirus* genus in the family *Phenuiviridae*. RVFV is present throughout Africa and the Middle East and causes important economic losses in livestock [6,25,88]. During an epidemic in 2012, human cases and deaths occurred in Mauritania [6]. *Culex* and *Aedes* spp. are proven vectors of this virus, but *An. arabiensis*, *An. coustani*, *An. rufipes*, *An. pharoensis*, *An. rhodesiensis*, and *An. christyi* have also been implicated in transmission during epizootics and epidemics, as well as in maintenance by vertical transmission [25].

#### 3.4.4. Peribunyavirus: Leanyer Virus (LEAV), Ngari Virus (NRIV), Bangui Virus (BGIV), Cache Valley Virus (CVV), Mapputta Virus (MAPV), Tahyna Virus (TAHV), Tataguine Virus (TATV), Batai Virus (BATV), Nyando Virus (NDV), Ilesha Virus (ILEV), Bwamba Virus (BWAV), *Anopheles* A Virus (ANAV), *Anopheles* B Virus (ANBV), Tensaw Virus (TENV)

At least 14 species of the genus *Orthobunyavirus* in the family *Peribunyaviridae* have been detected in *Anopheles* species. The orthobunyaviruses are characterized by a single-stranded RNA genome of negative polarity composed of three segments that are large (L), medium (M), and small (S) encoding the RNA dependent RNA polymerase, the glycoproteins (Gn and Gc) and the nucleoprotein, respectively [49].

Leanyer virus (LEAV) was isolated from *An. meraukensis* pools in a suburb of Darwin, Australia in 1974, and was cultivable on BHK-2I and Vero cells [48]. Peptide sequence analysis indicated that LEAV is related to Oropouche virus with 59% similarity in the polymerase sequence. LEAV does not cross-react serologically with other orthobunyaviruses and the L and S segment peptide sequences display divergence from other orthobunyaviruses, and therefore LEAV could be considered as a new antigenic complex [89].

Batai virus (BATV) was first isolated from *Culex gelidus* in Malaysia [90]. More recently, two different entomological surveys in Germany and Italy identified and isolated BATV from *Anopheles maculipennis* complex mosquitoes [20,37]. The Italian and German strains were related to strains isolated in Slovakia [37]. BATV causes hemorrhagic disease, with fever, headache, nausea, and vomiting. BATV was also identified in Sudan from sera of febrile patients [90]. Cattle are a potential host of BATV, and in a survey in Germany, the serological prevalence in cattle was 0.55%, while a 2.1% positivity rate was detected by RT-PCR in cattle from Mongolia [91,92]. The *An. maculipennis* complex is an important historical European vector of *P. vivax*, and a current vector in Europe and Asia [93].

Recombination events between BATV and Bunyamwera virus generated Ngari virus (NRIV), which has mainly been reported in Africa. The first isolation of NRIV was done from *Ae. simpsoni* in Senegal in 1979. It was also isolated from *An. gambiae* and *An. pharoensis*, and cytopathic effects were observed in Vero cells [36]. Pools of *An. funestus* collected in Kenya between 2007 and 2012 were positive for NRIV RNA by RT-PCR and sequencing. The full genome sequence of NRIV was obtained from mosquito and human samples [94].

Bangui virus (BGIV), a probable arbovirus of this family, was first isolated from humans in 1973 in the Central African Republic, where it was also detected in *An. pharoensis* [36]. BGIV produces cytopathic effects on Vero and amphibian *Xenopus* cells, and is sensitive to ether and acid pH [95]. Serological tests are used for the detection of BGIV, but molecular tools are lacking.

Cache Valley virus (CVV), in vector competence assays, was more infectious to *An. quadrimaculatus* than to *Coquillettidia perturbans*. At 16 d post-infection, infection prevalence was greater than 90% for both species [39]. Human and other vertebrate cases of CVV have been reported, including from a woman diagnosed with aseptic meningitis [96]. CVV can be cultured on many vertebrate cells such as Buffalo green monkey kidney (BGMK), human colon adenocarcinoma (CaCo2), human lung carcinoma (A549), and Vero cells.

Mapputta virus (MAPV) was isolated in 1960 from *An. meraukensis* in Australia. MAPV antibodies react with other viruses of Mapputta group such as Maprik virus (MPKV), Trubanaman virus (TRUV), and Gan Gan virus (GGV), indicating that a serological test is not sufficient to distinguish them [49]. There is at least 60% nucleotide identity of S and L segments between MAPV and MPKV [49]. *An. meraukensis* bites humans and other vertebrates, but it has not been incriminated as a malaria vector [97,98]. Mapputta virus can be cultured on hamster kidney BHK-21 cells, which display cytopathic effects 4 d post-inoculation [49].

Tahyna virus (TAHV) was isolated from pools of *Anopheles hyrcanus* females collected in South Moravia [55], although it has been more often found in *Aedes* spp. *An. hyrcanus* extract was inoculated intracerebrally into mice and the virus was identified by neutralization tests on Vero E6 cells and confirmation by RT-PCR. The virus was generally fatal to inoculated mice. TAHV infection causes human fever, conjunctivitis, pharyngitis, malaise, arthralgia, headache, and drowsiness, and anti-TAHV IgM antibodies were detected by IFA in asymptomatic patients in China, but no human deaths have been attributed to this virus [55,99,100]. The *An. hyrcanus* group is a widespread species group involved in the transmission of *P. vivax* and *P. falciparum* in Europe and Asia [101,102].

Tataguine virus (TATV) takes its name from the village in Senegal where it was first isolated in 1962 from *Anopheles* and *Culex* species [56]. In 1966, it was also isolated in Cameroon from *An. gambiae* and from serum of a 14-year-old boy. The patient presented with fever, exanthema, asthenia, muscle aches, and neutropenia, and TATV was confirmed by inoculation in mice [56]. TATV was widespread in African countries surveyed from 1960 to 1970, including Nigeria, South Africa, and Ethiopia [103,104].

Nyando Virus (NDV) was isolated by inoculation of mice with extract of *An. funestus* collected in Kenya during the ONNV outbreak of 1959–1960. [18,50]. Cytopathic effects of NDV were observed on Vero E6 and RE05 cells but not on C6/36. NDV displays at least 90% nucleotide and peptide identity with Bwamba virus (BWAV) and Pongola virus (PGAV) [105]. NDV causes moderate to severe febrile disease in humans, and human exposure was detected in serological surveys in Kenya, Central African Republic, and Uganda [18,19].

Ilesha virus (ILEV) was isolated from a pool of *An. gambiae* collected in the Central African Republic [44]. ILEV was recovered in 1990 from the blood of a woman who died with fever, anemia, leucopenia, and coagulative disorders. The virus was isolated after inoculation of mosquito extract into suckling mice and was cultured on Vero E6 and AP61 cells [106].

Bwamba virus (BWAV) was isolated from extract of *An. funestus* in Uganda and from a human blood sample from a refugee camp in Tanzania [38]. BWAV appears to be a widespread human infection in Africa, with short duration symptoms including fever, headache, exanthema, arthralgia, body rash, and diarrhea [44,107].

*Anopheles* A virus (ANAV) and *Anopheles* B virus (ANBV) were first isolated from female *An. boliviensis* in Colombia. Mice injected with ANAV and ANBV displayed central nervous system pathologies [30], although these viruses have not been isolated from naturally-infected vertebrates. *An. boliviensis* is a minor malaria vector in Colombia [108]. ANAV and ANBV are distinct from other Bunyamwera viruses because the S segment encodes only the nucleocapsid protein N, and therefore the nonstructural protein (NSs) involved in replication and pathogenesis is absent [109,110,111].

Tensaw virus (TENV) from Tensaw River in the southeastern United States was isolated from *An. crucians* and *An. quadrimaculatus* [57]. Humans, dogs, raccoons and cows were positive for TENV by serological tests, indicating that TENV was transmitted from mosquitoes to vertebrates. *An. quadrimaculatus* and *An. albimanus* remained infective from 2–14 d post-infection [57].

#### 3.4.5. Dicistrovirus: *Anopheles* C Virus (AnCV) and *Anopheles* Associated C Virus (AACV)

Two *Anopheles* dicistroviruses were identified by deep sequencing and de novo assembly. Anopheles associated C virus (AACV) [16] and Anopheles C virus (AnCV) [5] belong to the genus *Cripavirus* in the *Dicistroviridae* family, non-enveloped viruses with a single stranded RNA genome of positive polarity. The dicistrovirus genome is comprised of two open reading frames (ORF). ORF1 encodes the non-structural proteins necessary for virus replication, and ORF2 the viral proteins VPO to VP4. In the C-terminal region of ORF1, there is an RNA dependent RNA polymerase (RdRp) domain that is highly conserved among dicistroviruses [112]. Dicistroviruses are only known to infect insects.

AnCV is distinct from but related to Drosophila C virus, and was discovered in wild-caught human host-seeking *Anopheles* in Senegal, and in laboratory colonies of *An. coluzzii* in France and *An. dirus* in Cambodia [5]. Both horizontal and vertical modes of transmission were demonstrated in the Ngousso colony of *An. coluzzii*, while a colony of *Anopheles stephensi* maintained in the same laboratory as the *An. coluzzii* colony was not infected by AnCV [5].

AACV was discovered in *Anopheles maculipennis* collected in the Camargue region of France. AACV displays approximately 20% peptide sequence divergence from chronic bee paralysis virus (CBPV) in examined regions of the genome [16].

#### 3.4.6. Cypovirus: *Anopheles* Cypovirus (AnCPV)

Viruses in the genus *Cypovirus*, family *Reoviridae*, are characterized by a genome of 9 to 12 segments of linear dsRNA within a single capsid shell [113]. Cypoviruses, or cytoplasmic polyhedrosis viruses, are so named because the infectious forms are occlusion bodies in crystalline polyhedral form within the cytoplasm of infected cells. The protein polyhedra protect the virus against harsh environmental conditions such as high pH. Cypoviruses are only known to infect insects. The first *Anopheles* cypovirus was detected in adult *An. stephensi* by microscopy after staining with ammonium molybdate [41]. However, culture and transmission experiments did not succeed, the virus disappeared from the colony after nine months, and was not sequenced or named. This cypovirus was observed within the cytoplasm of *Plasmodium berghei* or *P. yoelii* rodent malaria oocysts in co-infected mosquitoes, and observations suggested it might have reduced the numbers of developing oocysts.

More recently, *Anopheles* cypovirus (AnCPV) was discovered in wild-caught human host-seeking *Anopheles* in Senegal and Cambodia, and was also present in the Ngousso laboratory colony of *An. coluzzii* [5]. AnCPV infection was absent in an *An. stephensi* colony maintained in the same laboratory.

#### 3.4.7. Orbivirus: Tibet Orbivirus (TIBOV), *Anopheles annulipes* Orbivirus (AAOV) *Anopheles hinesorum* Orbivirus (AHOV), Orungo Virus (ORUV), Tilligerry Virus (TILV)

Viruses of the *Orbivirus* genus, in the *Reoviridae* family, infect plants and vertebrates, and are characterized by a dsRNA genome of 10–12 segments. Tibet orbivirus (TIBOV) was isolated from *Anopheles maculatus* collected in China [58]. TIBOV was cultured from mosquito extract inoculated on C6/36 and BHK-21 cells. Cytopathic effects characterized by cell rounding, lysis, and floating cells were observed only in the BHK-21 cells after 3 d of infection, but viral RNA was detected by RT-PCR in both cell types. Gel electrophoresis revealed ten dsRNA genome segments, which were sequenced [58]. *An. maculatus* species are important vectors of human malaria in Asia [114].

Orbivirus sequences detected in *Anopheles annulipes* and *An. hinesorum* from Australia were named *Anopheles annulipes* orbivirus (AAOV) and *Anopheles hinesorum orbivirus* (AHOV) [31]. The viruses could not be cultured on C6/36 cells, but virus sequences were present in the mosquito 21-nucleotide viral RNA fraction, indicative of active replication and dicing of dsRNA replication intermediates.

Tilligerry virus (TILV) was isolated in 1971 from *An. annulipes* in Australia, and the complete genome sequence was determined [59]. The G + C content of the full genome of TILV is 45% and its 10 segments are visible on agarose and acrylamide gels [59,115]. TILV leads to cytopathic effects in BHK and BSR cells 2–3 d post inoculation [59]. TILV cross-reacts with bluetongue virus in complement fixation tests [115].

Orungo Virus was isolated in Uganda from *An. funestus* after inoculation on Vero and BHK-21 cells [51]. Viral replication was detected in the brains of inoculated mice and hamsters. Antibodies against ORUV were detected in human sera from Nigeria. The human symptoms of ORUV are fever, headache, myalgia, nausea, and vomiting, and ORUV also infects other vertebrates such as sheep, monkeys and cows [116]. On the basis of nucleotide G + C content and amino acid composition of the T2 protein, ORUV appears closer to *Culicoides*-borne than mosquito-borne orbiviruses [51].

#### 3.4.8. Mononegavirus: Bolahun Virus (BOAV) and Gambiae Virus (GAMV)

The order *Mononegavirales* are non-segmented and negative-sense single stranded RNA viruses encoding 5–10 ORFs. Sequences of two mononegaviruses were detected in *Anopheles* spp. in Liberia, Senegal, and Burkina Faso [32]. The two viruses, Bolahun virus (BOAV) and Gambiae virus (GAMV), have similar genome organization with six non-overlapping ORFs.

#### 3.4.9. Almendravirus: Coot Bay Virus (CBV)

Coot Bay virus in the *Almendravirus* genus belongs to the *Rhabdoviridae* family in *Mononegavirales* order [40]. CBV was isolated from *An. quadrimaculatus* mosquitoes collected in 2013 in Florida, USA [40]. CBV could be cultured in *Aedes* C6/36 cells, but not in mammalian BHK-21 and Vero cells [40]. CBV does not cause apparent illness or deaths in suckling mice. The virion has a diameter of about 50 nm [40].

#### 3.4.10. Totivirus: *Anopheles* Totivirus (AToV) and Australian *Anopheles* Totivirus (AATV)

A virus of the genus *Totivirus*, family *Totiviridae, Anopheles* totivirus (AToV) was discovered in *An. gambiae* from Liberia, with an infection prevalence of 1.3% [32]. Another totivirus, Australian *Anopheles* totivirus (AATV), was detected in *An. annulipes* and *An. hinesorum* collected in Australia [31]. Despite the lack of AATV replication in C6/36 cells, the presence of 21-nt viral RNA sequences was regarded as diagnostic of active virus replication in mosquitoes. AATV and AToV share ~25% identity at both nucleotide and peptide sequence levels. The genus *Totivirus* also includes protozoa-infecting members such as Trichomonasvirus, Victorivirus, Giardiavirus, and Leishmaniavirus [117]. This genus also commonly infects plants. A maize-associated totivirus was identified in China [118].

## 4. Discussion

Little is known about the *Anopheles* virome, as evidenced by the relatively small number of scientific publications on the topic. Nevertheless, when the literature summarized here is taken together, it is evident that *Anopheles* viruses, including among them pathogens of humans and other vertebrates, are abundant in nature but understudied.

We found published evidence of at least 51 viruses associated with *Anopheles.* This number is likely an underestimate, because it does not include publications in journals not indexed by the databases searched. The *Anopheles* virome appears to be dominated by RNA viruses. RNA viruses are also dominant in *Aedes* (Yellow fever virus, Zika virus, dengue virus, chikungunya virus) and *Culex* (Eastern equine encephalitis virus, Rift Valley fever virus, West Nile virus). RNA viruses evolve rapidly because of high mutation and recombination rates [119], and can potentially adapt rapidly to new hosts. There could also be an ascertainment bias favoring the detection of RNA as compared to DNA viruses, because sequencing of small viral-derived RNAs is a powerful tool to identify actively replicating RNA viruses [5,16,17].

It is likely that the main evolutionary pressure shaping mosquito antiviral mechanisms in general is their persistent exposure in nature to members of the natural virome, rather than the probably less frequent exposure to vertebrate-pathogenic arboviruses. Despite the apparently abundant presence of viruses in *Anopheles,* there is debate as to whether *Anopheles* mosquitoes serve as merely occasional hosts of pathogenic arboviruses, or to what extent they help mediate transmission as vectors. The distinction between host and vector will require evaluation of *Anopheles* vector competence in the laboratory and *Anopheles* vectorial capacity in the field. Vector competence is the ability to acquire, maintain, disseminate, and transmit a virus, whereas vectorial capacity or vector efficiency is the rate at which a putative vector population generates new inoculations from an infectious case.

The potential of the majority of *Anopheles*-associated viruses for transmission to humans or other vertebrates is currently unknown, because few studies of host range and transmission have been done. Some viruses may have a host range restricted to only *Anopheles* and other insects. For example, *Anopheles* cypovirus and *Anopheles* C virus were found to replicate and be maintained by vertical transmission in *An. coluzzii*, but were not able to infect *Ae. aegypti* in exposure experiments [5]. Both of these viruses were able to replicate in *An. stephensi* after exposure, but *Anopheles* C virus was not stably maintained and disappeared after several generations. Thus, these two viruses may be *Anopheles*-specific, but possibly not adapted to all *Anopheles* species.

A first group of viruses display either known or potential restriction of infection to *Anopheles* or insect cells. In some cases, detailed studies have demonstrated insect host restriction, while in other cases vertebrate cell infection or transmission potential has not yet been tested. This group includes *Anopheles gambiae* densovirus, Eilat virus, *Anopheles* flavivirus, *Anopheles gambiae* flavivirus, *Anopheles squamosus* flavivirus, Karumba virus, Haslams Creek virus, Dairy Swamp virus, Mac Peak virus, *Anopheles* C virus, *Anopheles* Associated C virus, *Anopheles* cypovirus, *Anopheles annulipes* orbivirus, *Anopheles hinesorum* orbivirus, Bolahun virus, Gambiae virus, Coot Bay virus, *Anopheles* totivirus, Australian *Anopheles* totivirus, Long Pine key virus, and Kampung Karu virus.

A different group of *Anopheles* viruses possesses likely vertebrate transmission potential, because studies have detected presence in vertebrates and/or replication in vertebrate cells. Further work will be required to confirm and characterize transmission between *Anopheles* species and vertebrates and evaluate their risk. This group of potential arboviruses includes *Anopheles minimus* virus, Leanyer virus, Tilligerry virus, Stratford virus, Ngari virus, Bangui virus, Mapputta virus, Nyando virus, Ilesha virus, Bwamba virus, Orungo virus, and *Anopheles* B virus.

Finally, known pathogenic arboviruses with evidence of presence in *Anopheles* include O’nyong nyong virus (ONNV), Venezuelan equine encephalitis virus, Western equine encephalitis virus, Sindbis virus, Semliki Forest virus, Rift Valley fever virus, West Nile virus, Japanese encephalitis virus, Wesselsbron virus, Tataguine virus, Batai virus, Cache Valley virus, Tahyna virus, and Tensaw virus. Myxoma virus can be mechanically transmitted by mosquitoes and fleas but does not replicate in them, and thus is not an arbovirus. Of these, transmission by *Anopheles* has been demonstrated for ONNV, but additional work is required to determine the vector competence and capacity of *Anopheles* for the other arboviruses.

The above grouping of viruses is likely to be porous and is expected to change with the addition of data from new studies. The interesting question remains, nevertheless, whether *Anopheles* are less efficient arbovirus vectors than *Aedes* and *Culex*, or are simply under-recognized as virus vectors. If the first case were true, that is, lower vector competence of *Anopheles* for arboviruses, then understanding the biological mechanisms leading to their general resistance to virus transmission would be important and could lead to novel tools to control arbovirus transmission by *Aedes* and *Culex* vectors. As the current systematic review indicates, *Anopheles* are not inherently resistant to virus replication. Although the natural virome data do not yet exist to make a numerical comparison with *Aedes* and *Culex*, there is no evidence that rates of natural carriage of viruses are substantially different between these mosquito genera.

Genetically encoded differences between mosquito species can interact with viral factors to influence host permissiveness and restriction. The protein nsP3 of ONNV influences host specificity of this virus to *Anopheles* as compared to *Aedes*, because substitution of chikungunya virus nsP3 by ONNV nsP3 in the chikungunya backbone allows chikungunya infection of *An. gambiae* [70]. In addition to interaction between viral factors and host cell proteins, differences in the small RNA regulatory pathways such as microRNAs and piwi-RNAs between *Culex*, *Aedes*, and *Anopheles* may also play a role in restricting host range [120].

The incidence of co-infection of malaria and arboviruses is probably underestimated in endemic areas. In a recent survey in Senegal, the frequency of human co-infection by *P. falciparum* malaria and arboviruses (dengue, yellow fever, Zika, chikungunya, Rift Valley fever) was close to 50% [121]. It is unknown whether members of the natural virome can influence malaria parasite or arbovirus infection and transmission by the vector, for example by stimulating or diverting mosquito basal immunity, or otherwise either promoting or diminishing superinfection.

One conclusion of this study is that the databases PubMed, Web of Science, Scopus, and Lissa can yield different results for the same specific area of research. The four bibliographic databases are complementary. PubMed and Lissa are free to access without registration, which is not the case for Scopus and Web of Science. Web of Science yielded the most results (58 articles), because it includes articles from many countries. However, PubMed contains the most recent articles. In contrast, the articles found in Lissa are in French and include older works, which can fill gaps not covered by other databases. The Arbocat Arbovirus Catalog indexes arboviruses or potential arboviruses harbored by arthropods, but for some of these viruses, the support is unpublished or no longer available.

Finally, it should be noted that a systematic automated search profile can only reveal reports that are indexed using informative combinations of terms. A limiting factor is thus the quality of index terms in the records. The search parameters presented here could perhaps be modified with additional systematic terms. However, search terms that might appear to be more precise may in fact generate less informative results and lead to diminishing returns. For example, searching of databases with [virus name + Anopheles], can generate large numbers of often low-quality results (e.g., [West Nile virus] + Anopheles generates 140 records). Such searches require increased levels of labor-intensive manual curation to identify on-target records. Systematic automated searches can be easily re-run. Moreover, the most important biological observation is made when a virus is first reliably reported in at least one *Anopheles* species. After that, finding the virus in other *Anopheles* is useful, but hardly surprising, while studies of the biology of host range restriction and mode of transmission of *Anopheles* viruses are sorely lacking.

## 5. Conclusions

This study is the first, to our knowledge, to present an overview of the published literature on the *Anopheles* virome. At least 51 viruses have been reported in *Anopheles* in almost all continents. The quantification and identification of the *Anopheles* virome is important for general understanding of microbiome diversity, for surveillance and prevention of emergence of unknown viruses, to understand the phenomenon of human malaria and arbovirus co-infection, and to study the antiviral and immune responses of *Anopheles* mosquitoes. The availability of next-generation sequencing and de novo assembly will likely continue to augment knowledge of *Anopheles* viruses, and more effort will be required to characterize their biology and public health risk.

## Figures and Tables

**Table 1:** Summary of reported *Anopheles* viruses with references. (Sortable Excel table available for download from Supplementary Materials).

Virus Name	Abbreviation	Virus Genus	Anopheles Species	References
Anopheles A virus	ANAV	Orthobunyavirus	*Anopheles boliviensis*	[30]
Anopheles annulipes orbivirus	AAOV	Orbivirus	*Anopheles annulipes*	[31]
Anopheles associated C virus	AACV	Cripavirus	*Anopheles maculipennis*	[16]
Anopheles B virus	ANBV	Orthobunyavirus	*Anopheles boliviensis*	[30]
Anopheles C virus	AnCV	Cripavirus	*Anopheles gambiae*	[5]
Anopheles cypovirus	AnCPV	Cypovirus	*Anopheles gambiae*	[5]
Anopheles flavivirus	AnFV	Flavivirus	*Anopheles *sp.	[32]
Anopheles gambiae densovirus	AgDNV	Densovirus	*Anopheles gambiae*	[33]
Anopheles gambiae flavivirus	AngFV	Flavivirus	*Anopheles gambiae*	[34]
Anopheles hinesorum orbivirus	AHOV	Orbivirus	*Anopheles hinesorum*	[31]
Anopheles minimus virus	AMIV	Iridovirus	*Anopheles minimus*	[35]
Anopheles squamosus flavivirus	AnsFV	Flavivirus	*Anopheles squamosus*	[34]
Anopheles totivirus	AToV	Totivirus	*Anopheles gambiae*	[32]
Australian Anopheles totivirus	AATV	Totivirus	*Anopheles annulipes*	[31]
Australian Anopheles totivirus	AATV	Totivirus	*Anopheles hinesorum*	[31]
Bangui virus	BGIV	Orthobunyavirus	*Anopheles pharoensis*	[36]
Batai virus	BATV	Orthobunyavirus	*Anopheles maculipennis*	[20,37]
Bolahum virus	BOAV	Mononegavirus	*Anopheles *sp.	[32]
Bwamba virus	BWAV	Orthobunyavirus	*Anopheles funestus*	[38]
Cache Valley virus	CVV	Orthobunyavirus	*Anopheles quadrimaculatus*	[39]
Coot Bay virus	CBV	Almendravirus	*Anopheles quadrimaculatus*	[40]
Cypovirus	Unnamed	Cypovirus	*Anopheles stephensi*	[41]
Dairy Swamp virus	DSwV	Flavivirus	*Anopheles bancrofti*	[42]
Eliat virus	EILV	Alphavirus	*Anopheles coustani*	[13,43]
Gambiae virus	GAMV	Mononegavirus	*Anopheles *sp.	[32]
Haslams Creek virus	HaCV	Flavivirus	*Anopheles annulipes*	[42]
Ilesha virus	ILEV	Orthobunyavirus	*Anopheles gambiae*	[44]
Japanese encephalitis virus	JEV	Flavivirus	*Anopheles peditaeniatus*	[45]
Japanese encephalitis virus	JEV	Flavivirus	*Anopheles sinensis*	[46]
Kampung karu virus	KPKV	Flavivirus	*Anopheles tesselatus*	[47]
Karumba virus	KRBV	Flavivirus	*Anopheles meraukensis*	[42]
Leanyer virus	LEAV	Orthobunyavirus	*Anopheles meraukensis*	[48]
Long Pine key virus	LPKV	Flavivirus	*Anopheles crucians*	[47]
Mac Peak virus	McPV	Flavivirus	*Anopheles farauti*	[42]
Mapputta virus	MAPV	Orthobunyavirus	*Anopheles meraukensis*	[49]
Myxoma virus	MYXV	Poxvirus	*Anopheles maculipennis*	[22]
Ngari virus	NRIV	Orthobunyavirus	*Anopheles gambiae*	[36]
Nyando virus	NDV	Orthobunyavirus	*Anopheles funestus*	[18,50]
O’nyong nyong virus	ONNV	Alphavirus	*Anopheles gambiae*	[8,50]
O’nyong nyong virus	ONNV	Alphavirus	*Anopheles funestus*	[8,50]
Orungo virus	ORUV	Orbivirus	*Anopheles funestus*	[51]
Rift Valley fever virus	RVFV	Phlebovirus	*Anopheles squamosus*	[25]
Rift Valley fever virus	RVFV	Phlebovirus	*Anopheles coustani*	[25]
Semliki Forest virus	SFV	Alphavirus	*Anopheles funestus*	[52]
Semliki Forest virus	SFV	Alphavirus	*Anopheles coustani*	[52]
Sindbis virus	SINV	Alphavirus	*Anopheles pharoensis*	[53]
Sindbis virus	SINV	Alphavirus	*Anopheles albimanus*	[53]
Stratford virus	STRV	Flavivirus	*Anopheles annulipes*	[54]
Tahyna virus	TAHV	Orthobunyavirus	*Anopheles hyrcanus*	[55]
Tataguine virus	TATV	Orthobunyavirus	*Anopheles gambiae*	[56]
Tensaw virus	TENV	Orthobunyavirus	*Anopheles crucians*	[57]
Tibet orbivirus	TIBOV	Orbivirus	*Anopheles maculatus*	[58]
Tilligerry virus	TILV	Orbivirus	*Anopheles annulipes*	[59]
Venezuelan equine encephalitis virus	VEEV	Alphavirus	*Anopheles pseudopunctipennis*	[60]
Wesselsbron virus	WSLV	Flavivirus	*Anopheles coustani*	[34]
West Nile virus	WNV	Flavivirus	*Anopheles pauliani*	[23]
West Nile virus	WNV	Flavivirus	*Anopheles maculipennis*	[61,62]
Western equine encephalitis virus	WEEV	Alphavirus	*Anopheles albitarsis*	[63]

## Supplementary Materials

The following are available online at http://www.mdpi.com/1999-4915/10/5/222/s1, Figure S1: Prisma Flow Diagram of search results and record filtering. Table S1, Summary of reported Anopheles viruses with references.
