# Supplementary material for: A Systematic Review of the Natural Virome of Anopheles Mosquitoes
Source: Viruses. 2018 Apr 25;10(5):222. doi: 10.3390/v10050222 (PMC5977215; doi:10.3390/v10050222)
Supplement: Supplementary file 1 [file viruses-10-00222-s001.zip › Figure S1 Prisma Flow Diagram of search results and record filtering.docx]

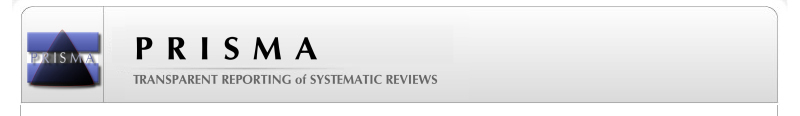
**PRISMA 2009 Flow Diagram**

Studies included in quantitative synthesis (meta-analysis)
(n = NA )

Studies included in qualitative synthesis
(n = 151)

Full-text articles excluded, with reasons
(n = 2 )

Full-text articles assessed for eligibility
(n = 153 )

Records excluded
(n = 23 )

Records screened
(n = 176)

Records after duplicates removed
(n = 176)

Additional records identified through other sources
(n = 121 )

a)

Identification

Eligibility

Included

Screening

Records identified through database searching
(n = 138 )
